# Spotlight on the real-world treatment of CML pts in Germany: a retrospective survey in private oncology practices

**DOI:** 10.1007/s00277-024-05702-2

**Published:** 2024-03-12

**Authors:** Georg-Nikolaus Franke, Gunnar Loewe, Marcel Reiser, Hartmut Linde, Andreas Josting, Eyck von der Heyde, Uwe Platzbecker, Rudolf Weide, Hans Tesch, Arndt Nusch, Jolanta Dengler, Kathleen Jentsch-Ullrich

**Affiliations:** 1 Department for Hematology, Cellular Therapies, Hemostaseology and Infectious Diseases, University of Leipzig Medical Center, Leipzig, Germany; 2 Novartis Pharma GmbH, Nuremberg, Germany; 3 PIOH-Zentrum Praxis Internistischer Onkologie und Hämatologie, Cologne, Germany; 4 MVZ für Blut- und Krebserkrankungen, Potsdam, Germany; 5 Schwerpunktpraxis für Onkologie, Gastroenterologie, Hämatologie und Palliativmedizin, Berlin, Germany; 6 Studienzentrum am Raschplatz GbR, Hannover, Germany; 7 Praxis für Hämatologie und Onkologie, Koblenz, Germany; 8 Centrum für Hämatologie und Onkologie Bethanien, Frankfurt, Germany; 9 MVZ-Onkologie Velbert/Ratingen GbR, Velbert, Germany; 10 Onkologische Schwerpunktpraxis Heilbronn, Heilbronn, Germany; 11 Gemeinschaftspraxis für Hämatologie und Onkologie, Magdeburg, Germany

**Keywords:** Chronic myeloid leukemia, real-world data, *BCR::ABL1*

## Abstract

Clinical trials in chronic myeloid leukemia (CML) are usually carried out in specialized centers whereas primary care for patients (pts) with CML is mainly provided by local oncology practices. The aim of this study was to assess treatment practices in pts with CML in the setting of private oncology practices in Germany. We collected data of 819 pts with a confirmed diagnosis (dx) of CML in 2013 or later from 43 practices. At dx, 84.2% (*n*=690) and 9.4% (*n*=77) of pts were in chronic or accelerated phase, 0.7% (*n*=6) had a blast crisis. Molecular monitoring was provided by EUTOS certified laboratories in 87.7% of pts. Typical *BCR::ABL1* transcripts were detected in 86.6% (*n*=709). Molecular response was assessed after 2.8, 6.0, 9.4 and 12.9 m (mean) after start of treatment. Of the pts with available data, 11.1% did not achieve early molecular response and at 18 m, 83.7% had at least a major molecular response. 288 (35.2%) of pts switched to 2^nd^ line (2L) treatment after a mean of 21.0 months. Reasons for 2L treatment were side effects in 43.4% and suboptimal response or failure in 31.4% of pts. 106 pts went on to third line (3L) treatment. 36.8 % of pts switched to and 92.8 % of pts still on 3L treatment achieved *BCR::ABL1*^IS^ ≤1% at 12 m. In conclusion, in Germany pts with CML are routinely monitored by qPCR and good responses are achieved in the majority. Treatment changes are mainly due to adverse events rather than suboptimal responses.

## Introduction

Chronic myeloid leukemia (CML) is a myeloproliferative disease characterized by hepatosplenomegaly and uncontrolled proliferation of leuko- and thrombocytes and a triphasic disease course [1]. The underlying pathomechanism is a fusion gene, *BCR::ABL1*, which encodes for a constitutionally active tyrosine kinase also called BCR::ABL1 [2]. In Germany, the median age at diagnosis is 65 years and slightly more patients are male (58%). Incidence and prevalence are age-dependent and are estimated to be 1.8/100.000/year and 14.9/100,000 for the German population, respectively [3].

Untreated, the disease is usually progressive within 3-5 years, leading to an acute leukemia like phenotype called blast crisis. Since the advent of tyrosine kinase inhibitors (TKI) at the end of the last millennium, treatment consists of one of the approved TKI’s resulting in a normal life expectancy in patients with CML [4]. Strict guidelines for diagnosis, treatment and monitoring of patients with CML have been published by the EuropeanLeukemiaNet since the early 2000s, with the most recent update published 2020, which were used in many of the CML trials [5–7]. Thus, the knowledge about the treatment reality of patients in Germany treated outside these large centers is limited [5, 8]. Treatment milestones nowadays are defined as certain molecular levels of the *BCR::ABL1* transcript as measured by RT-q-PCR or digital PCR against a reference gene (usually *ABL1*) normalized to the International scale (IS) to compensate for the high interlaboratory variability of the method [9–11]. The pivotal STIM trial and the confirmatory EURO-SKI trial showed that a proportion of 40-50% of the patients in deep molecular remission (MR4 (BCR-ABL1 %IS ≤0.01) or better) for a sufficient time can even discontinue treatment without loss of molecular remission [12, 13]. This concept also seems feasible in a real-world setting [14].

In Germany, the majority of data on CML stems from clinical trials in academic centers and specialized treatment units. Patients with cardiovascular and intermediate and severe chronic kidney disease are usually excluded, as well as patients with gastrointestinal, autoimmune or other relevant disorders. Despite this, TKI treatment will be administered to almost all patients with CML, regardless of existing comorbidities. In the real world most patients with CML are treated in private practices and many of these patients do not fulfil inclusion and exclusion criteria of the trials, resulting in a scarcecity of data in this setting. To close this knowledge gap, we aimed to retrospectively gather data on current treatment practices and outcomes in Germany outside academic centers and specialized treatment units.

## Objectives and methods

We aimed to systematically map the primary care of CML patients in Germany within the different treatment lines and to assess the proportion of patients showing a suboptimal course of therapy or therapy failure according to the 2013 ELN recommendations on CML under TKI treatment in the first, second and third line of therapy. Furthermore, we wanted to detect the proportion of patients who underwent at least one TKI therapy change, assess the routine therapy monitoring of CML patients at private practices and the therapeutic decision based on the line of therapy for CML patients who did not achieve an early molecular response (*BCR-ABL1*^IS^ > 10% after 3 months) under TKI therapy. To be included, patients had to have a newly diagnosed CML after 2012 and had to be treated at a specific center for at least 12 months and still in treatment or surveillance at the center at the time of data entry. The minimum requirement for a center to participate were more than 4 patients eligible for inclusion. Treatment was defined as having received a TKI, interferon or any other therapy except hydroxyurea. Anonymized data were gathered retrospectively from patient files by the participating center and aggregated per center to ensure patient anonymity. An independent vendor designed the eCRF, collected all reports and merged the aggregated data from an individual center into an overall cohort.

## Results

**Fig. 1:**
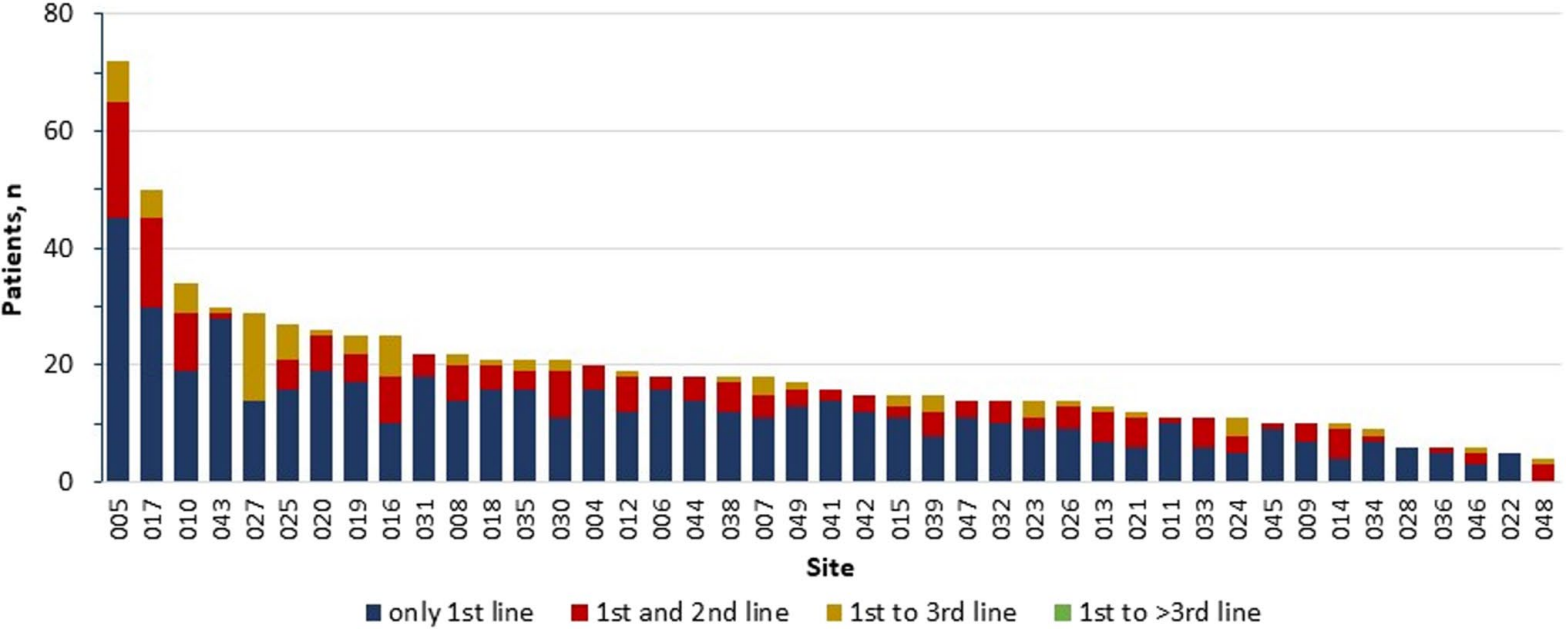
Distribution of patients and response by center

The 43 participating centers treated 1977 patients with newly diagnosed pts with CML between 2013 and 2022. The median number of patients per center was 35 (range 5 -280). 819 of these patients fulfilled the inclusion criteria and were entered into the survey. Median number of patients per center was 16 (range 5 – 75, Fig. 1). Mean age was 58.5 years (range 15 – 91) and 48.6% were female. 690 (84.3%) patients were in chronic phase (CP), 77 (9.4%) in accelerated phase (AP) and 6 (0.7%) were diagnosed in blast crisis (BC) (missing data in 46 (5.6%) patients). At diagnosis, palpable spleen size was documented in 503 (61.4%) patients and in 448 patients no risk score using any of established risk classifications (Sokal, EUTOS, ELTS or other) was calculated (Table 1). Using the documented data at the time of diagnosis, we were able to calculate EUTOS and ELTS scores for 460 and 457 patients, respectively. EUTOS risk was low in 89.6% (*n*=412) and high in 10.4% (*n*=48) of patients, whereas the ELTS score was low in 52.95% (*n*=242), intermediate in 32.2% (*n*=147) and high in 14.9% (*n*=68) of patients, comparable to another, smaller analysis regarding patients treated in German private oncology practices [15]. Molecular diagnostic regarding quantitative *BCR::ABL1* measurements was performed in a EUTOS certified laboratory in 718 (87.7%) patients, in 99 (12.1%) patients this diagnostic was done in a non-EUTOS certified lab (missing data for 2 patients). A typical *BCR::ABL1* transcript (e13/a2 or e14/a2) was detected in 672 (82.1%) patients, 39 (4.8%) had an atypical transcript and 37 (4.5%) of patients had both (data missing in 71 (8.7%) patients). *BCR::ABL1IS* at diagnosis was available in 692 patients and the mean *BCR::ABL1IS* was 46.2% (range 0 – 130). A mutation analysis of the *ABL1*-kinase domain at diagnosis was performed in 185 (22.6%) of the patients and was positive in 15 cases (1 V299L, 1 Y253H and 14 other mutations were detected). This is result is somewhat surprising, as nearly 85% of patients were in CP at the time of diagnosis, where a mutation analysis is not recommended by current and past ELN guidelines. Cytogenetics at diagnosis were documented for 588 (71.8%) patients, 218 patients did not have a cytogenetic analysis at diagnosis and data was missing in 13 patients. Mean percentage of Ph+ metaphases was 82.3% (range 0 – 100). Additional cytogenetics abnormalities were present in 79 of these patients (Table 1).

**Table 1:** Patients characteristics and risk scores at diagnosis

Number of patients	819
Sex, *n* (%)	female 398 (48.6) / male 421 (51.4)
Age at Dx, y (mean, range)	58.5 (15 -91)
ELN2013 disease phase at Dx, *n* (%)	chronic Phase 690 (84.2), accelerated Phase 77 (9.4), blast crisis 6 (0.7), missing 46 (5.6)
Spleen palpation done at dx, *n* (%)	yes 503 (61.4) / no 316 (38.6)
Risk score at Dx calculated at treatment center, *n* (%)	none or missing 476 (58.1) Hasford 159 (19.4) / Sokal 165 (20.1) / EUTOS 295 (36.0) / ELTS 29 (3.5) / other 2 (0.2)
Calculated scores by investigator, *n* (%)	EUTOS: low 412 (50.3) / high 48 (5.9%) / missing 359 (43.8) ELTS: low 242 (29.6)/ intermediate 147 (17.9)/ high 68 (8.3) / missing 362 (44.2)
Type of *BCR::ABL1* transcript, *n* (%)	e13/a2 or e14/a2 672 (82.1), atypical 39 (4.8), both 37 (4.5), unknown 71 (8.7)
Cytogenetics at Dx, *n* (%)	yes 588 (71.8) / no 218 (26.6) / missing 13 (1.6)
ACA at Dx, *n* (%)	79 of 588 (13.4)
Type of ACA at Dx, *n* (%)	Additional Ph+ 28 (35.4), Trisomy 8 9 (11.4), del(7) 2 (2.5), Chomosome 3 abnormalities 1 (1.3), trisomy 19 1 (1.3), other 38 (48.1)
Hydroxyurea pre-treatment	yes 275 (33.6) / no 529 (64.6) / missing 15 (1.8)

*ACA* additional cytogenetic abberations, *Dx* diagnosis, *ELTS* European Longterm Survival score, *y* years

**Fig. 2:**
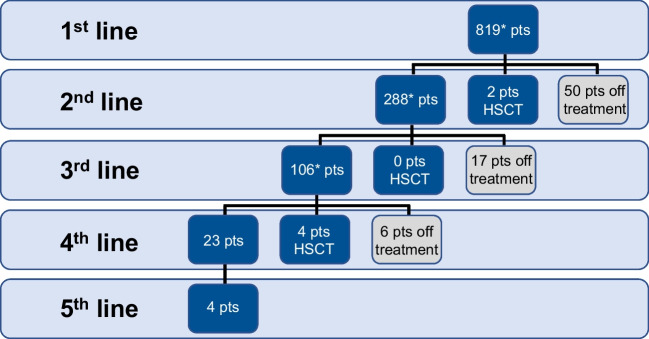
Patient disposition. Note: Patients remaining on particular treatment lines are not shown. *Patients entering a clinical trial - 1^st^ line: 154 (18.8%), 2^nd^ line: 7 (2.4%), 3^rd^ line 2 (1.9%). HSCT: hematopoietic stem cell transplantation; pts: patients

Of the 819 patients, 275 (33.6%) had a cytoreductive pre-treatment with hydroxyurea before starting the first line of therapy. 288, 106, 23 and 4 patients had to switch to 2nd, 3rd, 4th and 5th line treatment, respectively (Fig. 2). Mean treatment duration was 21 (range, 0 – 103) months in 1L, 17.6 (range, 0 – 97) in 2L and 16.4 (range, 0.26 – 80.5) months in 3L treatment. A total of 73 patients discontinued the treatment after 1st (*n*=50), 2nd (*n*=17) or 3rd line (*n*=6) therapy, whereas 6 patients underwent allogeneic hematopoietic stem cell transplantation (2 after 1st line and 4 after 3rd line treament). 163 patients (154 (18.8%) 1st, 7 (2.4%) 2nd line and 2 (1.9%) in 3rd line, respectively) were included into a clinical trial. Referral to an academic center was rare but increased with lines of therapy (at 1st, 2nd and 3rd line: 28 (3.4%), 10 (3.5%) and 8 (7.5%) patients).

Mean time from diagnosis to initiating 1st line treatment was 1.23 months (range 0 – 92.28). 215 patients had a cytogenetic response assessment after a mean of 5.75 months with 73.0% (*n*=157) complete cytogenetic responses, 17.2% (*n*=37) partial cytogenetic responses, 2.8% (*n*=6) minor cytogenetic responses and 2.8% (*n*=6) minimal cytogenetic responses. 9 (4.2%) patients did not have a cytogenetic response. A 2nd and 3rd cytogenetic analysis was done in 56 and 22 patients at a mean time of 10.0 and 13.6 months after start of 1st line therapy. Additional cytogenetic abnormalities were detected in 71 (8.7%) patients: 29 additional Ph+-chromosomes, 9 trisomy 8, 1 trisomy 19, 2 del(7), 2 aberrations on chromosome 3 and 40 other chromosomal aberrations.

Molecular response was assessed after a mean of 2.8, 6.0, 9.4, 12.9, 16.5, 19.9 and 23.6 months after initiating therapy in the first 2 years, although the range was high. For example, the range for the 3rd molecular response assessment was 1 – 62 months, reflecting that some patients did not have any monitoring while others were on a rather tight schedule. This did not change when patients switched to 2nd or later lines of treatment, where molecular monitoring was also performed roughly every 3 months when looking at the mean time since switching.

**Fig. 3:**
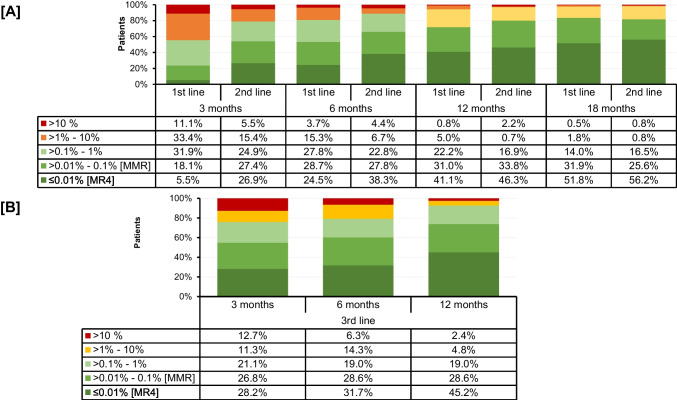
Molecular response according to time point and treatment line. (**A**) 1^st^ and 2^nd^ line (**B**) 3^rd^ line. Data based on non-missing | N (1^st^ line [3m; 6m; 12m; 18m]) = 623; 543; 504; 392 / N (2^nd^ line[ 3m; 6m; 12m; 18m]) = 201; 180; 134; 121 | N (1^st^ line [3m; 6m; 12m]) = 71; 63; 42

In 1st line, 11.1% did not achieve early molecular response (<10% *BCR::ABL1*^*IS*^) after 3 months. After 6 months, 3.7% of patients still on 1st line therapy had >10% *BCR::ABL1*^IS^. At 12 months, 5.8% of patients did not meet the ELN2013 milestones of ≤0.1% *BCR::ABL1* ^*IS*^, considered a “safe haven” for CML patients [16]. When looking at 2nd and 3rd line therapy, the proportion of patients with molecular failure (>1% *BCR::ABL1*^*IS*^) after 12 months of treatment was 2.9 and 7.2% (Fig. 3).

**Fig. 4:**
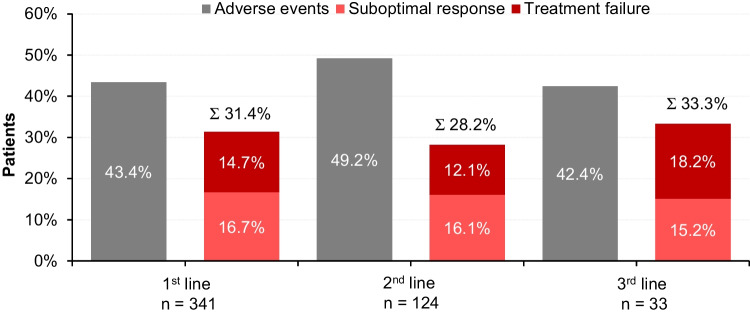
Reasons for switching treatment

**Fig. 5:**
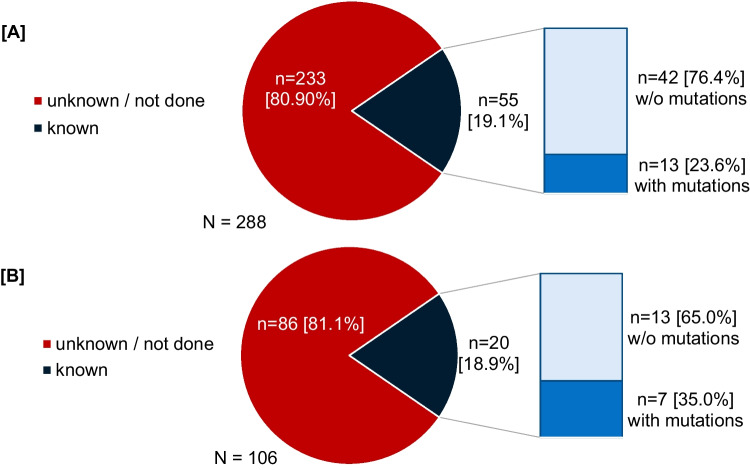
Mutation analysis of the ABL1-kinase domain and treatment failure. **A** at switch to 2^nd^ line (**B)** at switch to 3^rd^ line

As described above, 288, 106, 23 and 4 patients went on to 2nd, 3rd, 4th and 5th line of therapy. Reasons for switching treatment were adverse events in about 40% (1st line 43.3%, 2nd line 49.2%, 3rd line 42.4%), suboptimal response or molecular failure in 30% (1st line 31.4%, 2nd line 28.2%, 3rd line 33.3%) and other reasons in the remaining patients (Fig. 4). Mutational analysis of the *ABL1*-kinase domain was performed in a much smaller proportion of the patients (at switch to 2^nd^ line: 19.1%, at switch to 3rd line: 18.9%, at switch to 4^th^ line: 13.0%, Fig. 5). Cytogenetic analysis was done in 41 (14.2%) and 15 (7.1%) patients before 2^nd^ or 3^rd^ line therapy was initiated.

Looking at the distribution of the patients between the centers (Fig. 1), we noticed a significant skew in the number per center. The median number of patients treated was 19 and the ten biggest centers treated more than 40% of all patients included in the trial. Practices treating 19 or more CML patients were more likely to document the palpable spleen size (66.5 vs. 33.5%), assess a specific risk score (54.7 vs. 30.6%) and less likely to perform an ABL-Kinase domain mutation analysis (18.6 vs. 28.8%) at diagnosis. Despite this, patterns of cytogenetic and molecular monitoring, the proportion of patients still in 1^st^ line therapy after 12 months in MMR or better (72.7 vs. 70.9%) or switching to further lines of therapy (44.9 vs. 36.1%) were not different between centers treating more than the median numbers of patients compared to those treating less than the median.

## Discussion

The total number of patients with CML treated in the participating centers was more than 1900, representing at least 10% of patients in CML in Germany according to the prevalence in Germany, allowing a meaningful analysis of the treatment reality in Germany [3]. The patient characteristics at diagnosis are somewhat different from the large phase 3 trials: the patients in our cohort were older, and the gender distribution was almost even, compared to a predominance of male participants in most of the trials. In contrast to Scandinavian register data, less patients were in CP (84% vs 93%) at the time of diagnosis [17]. The rate of cytogenetic diagnostic prior start of therapy was only 71% in our cohort, clearly not in line with earlier and current ELN guidelines [5, 8]. In contrast, centers had strict molecular monitoring schedule: almost 90% of patients had molecular monitoring in a EUTOS certified laboratory and the mean time between molecular response assessment was about three months, regardless of the line of therapy. The rate of patients switching therapy at least once was comparable to an analysis by Kohlbrenner et al reporting on 124 patients of whom the 1^st^ line therapy was available from the InGef research database [3]. They found an incidence and prevalence of CML in Germany of 1.8 and 14.9 per 100,000 inhabitants. Most patients in this cohort received imatinib (42%), followed by nilotinib (35%) and dasatinib (10%). 26% of these patients switched therapy at least once. In our cohort, mutational analysis prior to changing therapy was performed in approximately 19% at switch to 2^nd^ and 3^rd^ line and Cytogenetics before switching treatment were analyzed in 14% and 7% before changing treatment to 2^nd^ and 3^rd^ line, respectively. The reason for changing therapy were adverse events in about 40-50% of patients, followed by lack of response in approximately 30% regardless of the line of therapy, meaning that a considerable number of patients did not receive appropriate diagnostic work up to identify mutations in the *ABL1* kinase domain or new cytogenetic aberrations indicating a progressive disease. As mutation analysis, at least by Sanger sequencing, was widely available even in 2013 and most practices reported to send their samples to an EUTOS certified laboratory, the reasons for this fact remain unknown. Weide et al reported earlier on a cohort of 248 patients with CML treated in private practices in Rhineland-Palatine [18]. 98% of these patients were diagnosed in chronic phase. The t(9;22) or the *BCR::ABL1* transcript were detected by cytogenetic analysis in 87% (or fluorescence in-situ hybridisation in 60%), whereas PCR-analysis was performed in 79%. They reported a rather low rate of molecular monitoring of 66% in patients within the first 12 months after diagnosis. 77% of these patients achieved an MMR or better after 12 months, comparable to the results in our cohort. 5% (*n*=12) of patients were transplanted compared to only 0.7% (*n*=6) of patients in this study and 9% in the German CML IV study [19].

The retrospective study has several limitations: due to the anonymized data necessary for data protection we do not have a longitudinal follow up on a single patient level, resulting in a lack of data on the patterns of TKIs use or other treatment modalities, treatment free remission and molecular response rates on a patient level in this cohort. It also prevents us from analyzing median durations of treatment per group and limits us to use the mean values. For the same reasons, we were not able to identify the TKI used, as there is only one 1^st^ and one 3^rd^ generation TKI. Especially in practices with a small number of CML patients, this would potentially allow the identification of patients. All depicted molecular responses per treatment line reflect only those patients, who were still on a specific line of treatment and had a response assessment. For example, at 18 months of 1^st^ line treatment, only 389 of 819 patients had remained on the same treatment and had had a response assessment. With this limitations, the molecular response rates seem to be similar to those reported by the other German cohorts and the large phase 3 trials on the first line use of TKI in CML [15, 20–23].

Limited by the restrictions imposed by the necessity to protect individual patient data, we were also not able to gather data on comorbidities and the type of adverse events leading to a switch of therapy or to estimate overall or progression free survival. Despite this, we were able to gain meaningful insight into today’s diagnosis, treatment, and monitoring practices for patients with CML in Germany in the last 10 years.

In conclusion, real-world treatment of CML patients is largely consistent with current guidelines, especially regarding adequate molecular monitoring. Although limited by the design of the study in following individual patients, switching therapy in patients with molecular failure or suboptimal response was not done in all cases and mutation analysis of the *ABL1*-kinase domain and cytogenetic work up as recommended by the ELN2013 guidelines was performed in less than half of the eligible patients. Interestingly, the main reason for a change in treatment was not resistance but intolerance, highlighting the fact that more specific inhibitors could further improve treatment results. Treatment outcomes were generally comparable to those published in the large phase 3 trials and other population-based analysis.

## References

[1] Minot GR, Buckman TE, Isaacs R (1924). Chronic myelogenous leukemia. J Am Med Assoc.

[2] Mauro MJ, O’Dwyer M, Heinrich MC, Druker BJ (2002). STI571: a paradigm of new agents for cancer therapeutics. J Clin Oncol.

[3] Saußele S, Kohlbrenner K, Vogelmann T, Schubert T (2022). Incidence, prevalence, and real-world treatment patterns in chronic myeloid leukemia: results from a population-representative german claims data analysis. Oncol Res Treat.

[4] Bower H, Björkholm M, Dickman PW (2016). Life expectancy of patients with chronic myeloid leukemia approaches the life expectancy of the general population. J Clin Oncol.

[5] Baccarani M, Deininger MW, Rosti G (2013). European LeukemiaNet recommendations for the management of chronic myeloid leukemia: 2013. Blood.

[6] Baccarani M, Saglio G, Goldman J (2006). Evolving concepts in the management of chronic myeloid leukemia: recommendations from an expert panel on behalf of the European LeukemiaNet. Blood.

[7] Hochhaus A, Baccarani M, Silver RT (2020). European LeukemiaNet 2020 recommendations for treating chronic myeloid leukemia. Leukemia.

[8] Hochhaus A, Baccarani M, Silver RT et al European LeukemiaNet 2020 recommendations for treating chronic myeloid leukemia. Leukemia 28:32. 10.1038/s41375-020-0776-2 10.1038/s41375-020-0776-2 PMC7214240 32127639

[9] White HE, Salmon M, Albano F (2022). Standardization of molecular monitoring of CML: results and recommendations from the European treatment and outcome study. Leukemia.

[10] Franke G-N, Maier J, Wildenberger K (2020). Comparison of real-time quantitative PCR and digital droplet PCR for BCR-ABL1 monitoring in patients with chronic myeloid leukemia. J Mol Diagn.

[11] Alikian M, Whale AS, Akiki S (2017). RT-qPCR and RT-digital PCR: A comparison of different platforms for the evaluation of residual disease in chronic myeloid leukemia. Clin Chem.

[12] Saussele S, Richter J, Guilhot J (2018). Discontinuation of tyrosine kinase inhibitor therapy in chronic myeloid leukaemia (EURO-SKI): a prespecified interim analysis of a prospective, multicentre, non-randomised, trial. Lancet Oncol.

[13] Mahon F-X, Réa D, Guilhot J (2010). Discontinuation of imatinib in patients with chronic myeloid leukaemia who have maintained complete molecular remission for at least 2 years: the prospective, multicentre Stop Imatinib (STIM) trial. Lancet Oncol.

[14] Dengler J, Tesch H, Jentsch-Ullrich K (2022). Treatment-free remission in real-world chronic myeloid leukemia patients: insights from german hematology practices. Acta Haematol.

[15] Weide R, Rendenbach B, Grundheber M (2017). Standard of care of patients with Chronic Myeloid Leukemia (CML) treated in community based oncology group practices between 2001-2015 in Rhineland-Palatinate (Germany). Appl Cancer Res.

[16] Hughes TP, Branford S (2009). Monitoring disease response to tyrosine kinase inhibitor therapy in CML. Hematology Am Soc Hematol Educ Program.

[17] Höglund M, Sandin F, Hellström K (2013). Tyrosine kinase inhibitor usage, treatment outcome, and prognostic scores in CML: report from the population-based Swedish CML registry. Blood.

[18] Weide R, Rendenbach B, Grundheber M (2017). Standard of care of patients with Chronic Myeloid Leukemia (CML) treated in community based oncology group practices between 2001-2015 in Rhineland-Palatinate (Germany). Appl Cancer Res.

[19] Hehlmann R, Lauseker M, Saußele S (2017). Assessment of imatinib as first-line treatment of chronic myeloid leukemia: 10-year survival results of the randomized CML study IV and impact of non-CML determinants. Leukemia.

[20] Hochhaus A, Saglio G, Hughes TP (2016). Long-term benefits and risks of frontline nilotinib vs imatinib for chronic myeloid leukemia in chronic phase: 5-year update of the randomized ENESTnd trial. Leukemia.

[21] Cortes JE, Saglio G, Kantarjian HM (2016). Final 5-year study results of DASISION: The dasatinib versus imatinib study in treatment-Naïve chronic myeloid leukemia patients trial. J Clin Oncol.

[22] O’Brien SG, Guilhot F, Larson RA (2003). Imatinib compared with interferon and low-dose cytarabine for newly diagnosed chronic-phase chronic myeloid leukemia. N Engl J Med.

[23] Saußele S, Kohlbrenner K, Vogelmann T, Schubert T (2022). Incidence, prevalence, and real-world treatment patterns in chronic myeloid leukemia: results from a population-representative german claims data analysis. Oncol Res Treat.

